# Supplementary material for: Epigenetic DNA Modifications Upregulate SPRY2 in Human Colorectal Cancers
Source: Cells. 2021 Oct 2;10(10):2632. doi: 10.3390/cells10102632 (PMC8534322; doi:10.3390/cells10102632)
Supplement: Supplementary file 1 [file cells-10-02632-s001.zip › Suppl Fig S1.pdf]

Supplementary Figure S1. MethPrimer: Bisulfite Primer Design For *SPRY2*

Region #1: Promoter: putative CTCF binding site [Chr13: 80,917,300-80,917,500]
Region #2: Promoter: upstream of the TSS [Chr13: 80,916,900-80,917,100]

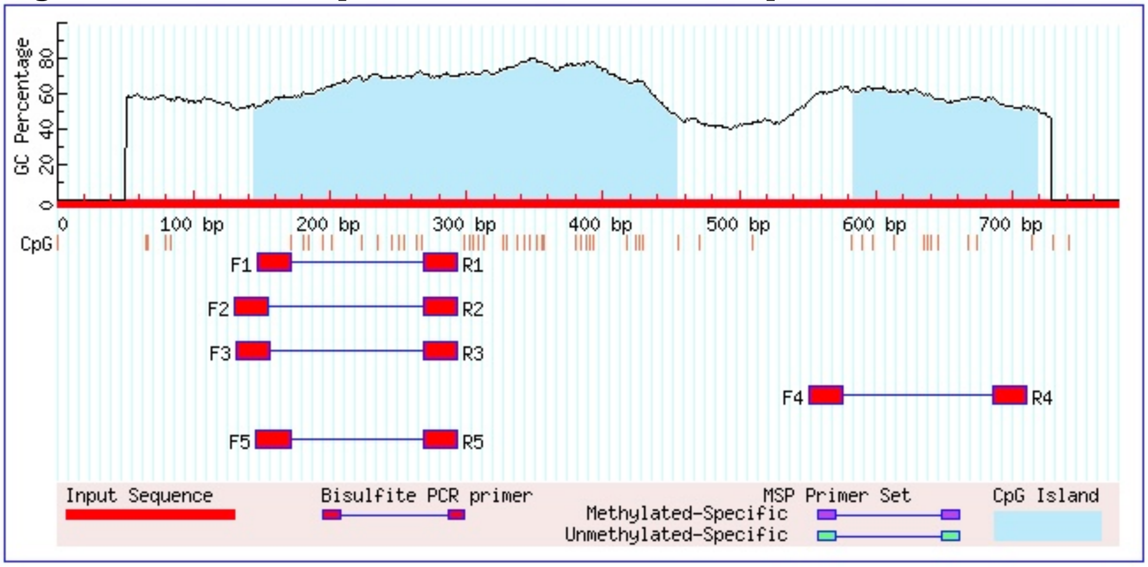

	Primer		Start	Size	TM	GC%	'C's	Sequence
	Left primer		551	25	57.93	56.00	5	TTAAAGTTTGGGTGTGTAAGGTTAG
Region #1: F4/R4	Right primer		710	25	57.74	56.00	7	AAAACCAACAAAACACAAATATCTC
	Product size: 160, Tm: 65.9, CpGs in product: 10							
	Left primer		147	25	58.60	56.00	4	TTAGAGATGGTAAGGGAAGTGGTAT
Region #2: F1/R1	Right primer		293	25	58.11	64.00	9	AACCAAAAAAACCCAACTATTTTAC
	Product size: 147, Tm: 65.0, CpGs in product: 12							

Region #3: Promoter: closest to the TSS [Chr13: 80,915,550-80,915,800]

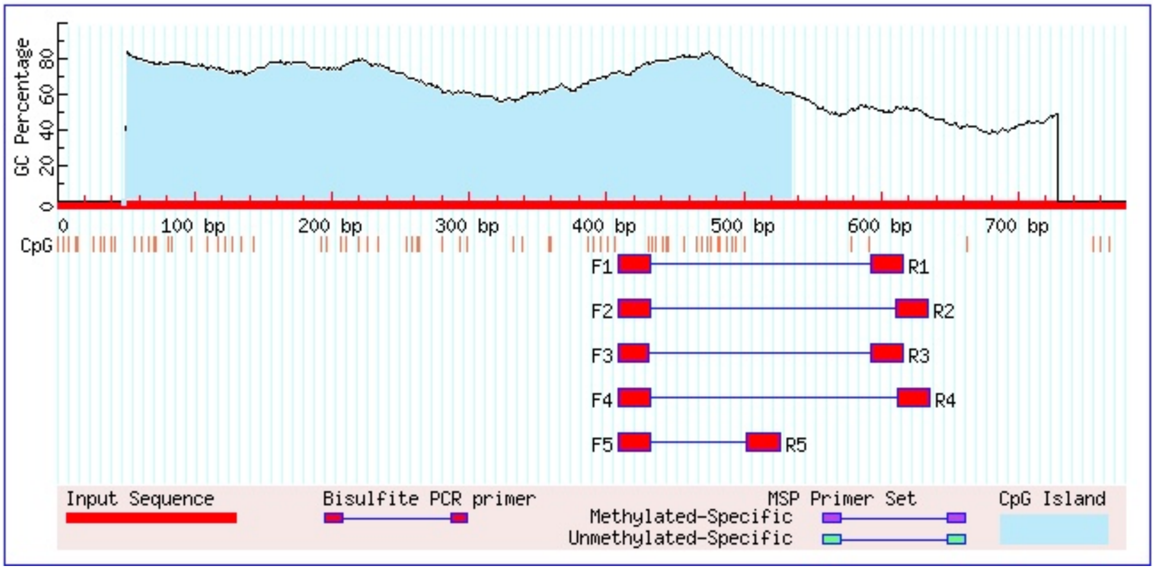

	Primer		Start	Size	TM	GC%	'C's	Sequence
	Left primer		408	24	53.60	54.17	5	GTAGGAGGTATTTAAAAGGGTAAT
Region #3: F1/R1	Right primer		616	24	56.35	50.00	6	AAAAAAAATTCCCTAAATCAACTC
	Product size: 209, Tm: 83.9, CpGs in product: 19							

Region #4: Intragenic CpG island: putative CTCF binding site [Chr13: 80,913,300-80,913,500]

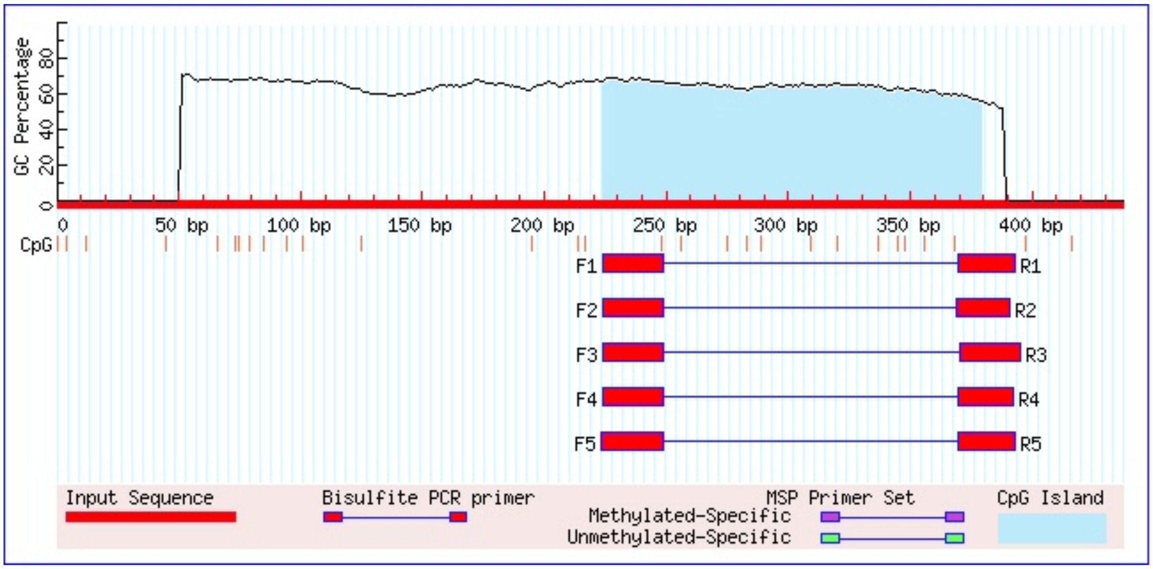

Region #4: F1/R1

Primer	Start	Size	TM	GC%	'C's	Sequence
Left primer	224	25	55.10	60.00	10	TTGTTAGGTTTTTTGTAAAGTTTTT
Right primer	393	24	59.89	54.17	4	CAAATTACAAACAATACCCCCTTC

Product size: 170, Tm: 69.9, CpGs in product: 12
