# Supplementary material for: Epigenetic DNA Modifications Upregulate SPRY2 in Human Colorectal Cancers
Source: Cells. 2021 Oct 2;10(10):2632. doi: 10.3390/cells10102632 (PMC8534322; doi:10.3390/cells10102632)
Supplement: Supplementary file 1 [file cells-10-02632-s001.zip › Suppl Fig S2.pdf]

UCSC Genome Browser depiction of SPYR2 in HCT116 and Caco2 cells

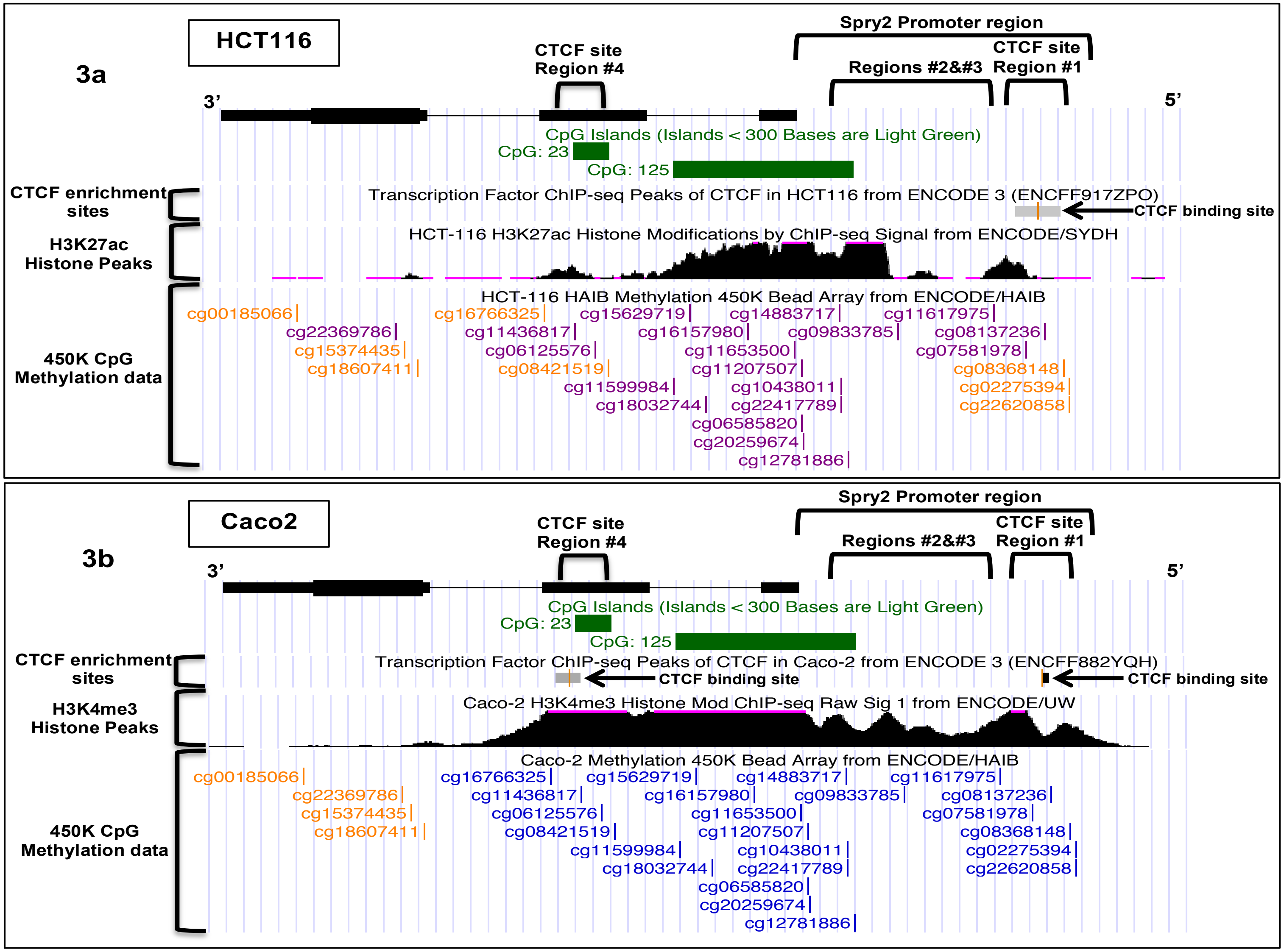

Supplementary Figure S2. UCSC Genome Browser (hg19 build) depiction of *SPRY2* in two CRC cell lines: (**a**) HCT116 and (**b**) Caco2. *SPRY2* contains 2 CpG islands (green) and according to ChIP-seq data, CTCF transcription factor binding sites in region #1 and #4 (grey bars=lower binding enrichment compared to solid black bars=highest binding enrichment). Note the binding of CTCF in region #1 in both CRC cell lines and also in region #4 in Caco2 cells. Also note the extensive methylation that is present in all 4 regions of *SPRY2* in HCT116 cells as indicated by purple (partial methylation) and orange (full methylation) vertical tick marks. In contrast, Caco2 cells contain no methylation spanning all 4 regions as indicated by blue (non-methylation) vertical tick marks. Lastly, ChIP-seq peaks associated with active transcriptional regulatory regions of *SPRY2* are represented by histone markers (H3K27ac) and (H3K4me3) in CRC cell lines HCT116 and Caco2, respectively.
